# Nutritional management of a dog with hepatic enzymopathy suspected to be secondary to copper-associated hepatitis: a case report

**DOI:** 10.3389/fvets.2023.1215447

**Published:** 2023-12-11

**Authors:** Francisco Manuel Poblanno Silva, Caitlin Elizabeth Grant, Érico de Mello Ribeiro, Adronie Verbrugghe

**Affiliations:** Department of Clinical Studies, Ontario Veterinary College, University of Guelph, Guelph, ON, Canada

**Keywords:** clinical nutrition, dietary copper, liver disease, homemade diet, alanine transaminase, hepatoprotective nutrients, liver biopsy, canine

## Abstract

A 4-year-old, female-spayed American Bulldog presented to the Ontario Veterinary College’s Health Sciences Center’s Clinical Nutrition Service for nutritional management of hepatic enzymopathy and suspected copper-associated hepatitis. Medical history revealed a 3-month history of gradually increasing serum ALT. Additional diagnostics included negative *Leptospira* titters, normal bile acids, and laparoscopic liver biopsy. Histopathology findings were consistent with diffuse moderate vacuole hepatocellular degeneration, mild positive copper staining, mild chronic lymphoplasmacytic hepatitis both portal and central, and mild biliary hyperplasia. Hepatic copper quantification results were above normal ranges (630 μg/g dry tissue), but below those seen in familial copper-associated hepatitis (>800–1,000 μg/g dry tissue). The patient was prescribed ursodeoxycholic acid, recommended to be fed a homemade diet (HMD), and referred for a nutrition consult. Two days before the nutrition consult, serum ALT fell within the normal range. The body condition score was 5/9, with a good muscle condition score and the dog’s appetite and body weight remained stable. Energy intake was appropriate for maintenance. Key nutrient levels of all diets reported were compared to industry standards (AAFCO, NRC, and FEDIAF). Diet history included a commercially available raw meat-based diet (RMBD), of unknown copper content; a high energy commercial dry food (HEC), with copper content higher than the maximum recommended by FEDIAF and immediately prior to nutrition consult the patient had been eating an unbalanced homemade diet (HMD1) for 4 weeks. HMD1 was low in copper and deficient in the hepatoprotectant nutrients vitamin E and zinc. As per the owner’s preference and to accommodate the patient’s unique nutritional needs, a homemade diet addressing key nutrients for liver disease was formulated (HMD2), with copper content just above the recommended minimum. The new diet was found palatable and the patient’s body weight, body, and muscle condition scores remained unchanged. Two months after starting HMD2, all bloodwork values remained within the normal range, including ALT. The reduction of dietary copper content likely reduced serum ALT. However, unbalanced diets cause a risk of nutrient deficiencies and excess. This dog was maintained with no known adverse effects on a complete and balanced HMD diet with a moderately low copper content, moderate protein, and inclusion of hepatoprotective nutrients.

## Background

Copper (Cu) is considered an essential dietary nutrient by all published pet food industry standards, National Research Council (NRC), American Association of Food Control Officers (AAFCO), and the European Pet Food Industry Federation (FEDIAF) (1–3). It is necessary for multiple metabolic processes as a cofactor for multiple enzymatic reactions, including mitochondrial respiration, erythropoiesis, protection against free radicals, neurotransmitter synthesis, pigmentation, and iron metabolism (4–6). Excessive accumulation of Cu could result in an increase in oxidative stress in the hepatocytes, consequently leading to cell damage and inflammation (7). Hepatic accumulation of Cu was first described in pure-breed dogs, such as Bedlington Terriers (8) and Labradors (9) among other dog breeds (10). The pathophysiological origin of this mineral accumulation was attributed to a genetic impairment in the Cu excretion from the hepatocytes (7). Without a genetic component, Cu accumulation could be secondary to cholestatic liver disease (11). According to the 2019 consensus statement of the American College of Internal Medicine on the diagnosis and treatment of chronic hepatitis in dogs, Cu is considered the most common toxic injury causing chronic hepatitis (12). Recent reports have questioned this finding and called for a revision of the current guidelines for Cu content in pet foods, suggesting an association with an increased incidence of Cu-associated hepatitis in dogs (12, 13). Regulatory officials recently responded by stating that Cu guidelines would remain unchanged while agreeing to a lack of evidence linking Cu content in foods with Cu-associated hepatitis (14). Nutritional management of Cu-associated hepatitis usually includes Cu restriction and supplementation of zinc and vitamin E (9, 11).

This case report presents a patient with elevated serum alanine transaminase (ALT) activity suspected to be secondary to Cu-associated hepatitis, who was previously fed a commercial raw meat-based diet (RMBD),^1^ a high-energy commercial extruded diet (HEC),^2^ and an unbalanced homemade diet (HMD1). The goal of the nutritional management was to prevent further liver damage while providing a complete and balanced diet. This case diet history includes one diet in which the Cu content was higher than the legal limit established by FEDIAF and one raw meat-based diet that had no nutritional adequacy statement and lacked a nutrient analysis, potentially exposing the dog to nutritional deficiency or excess. Lastly, the patient was eating a homemade cooked diet, which presented multiple nutritional deficiencies, including key nutrients that are commonly used for the management of liver disease (9, 11).

## Case presentation

**Figure 1:**
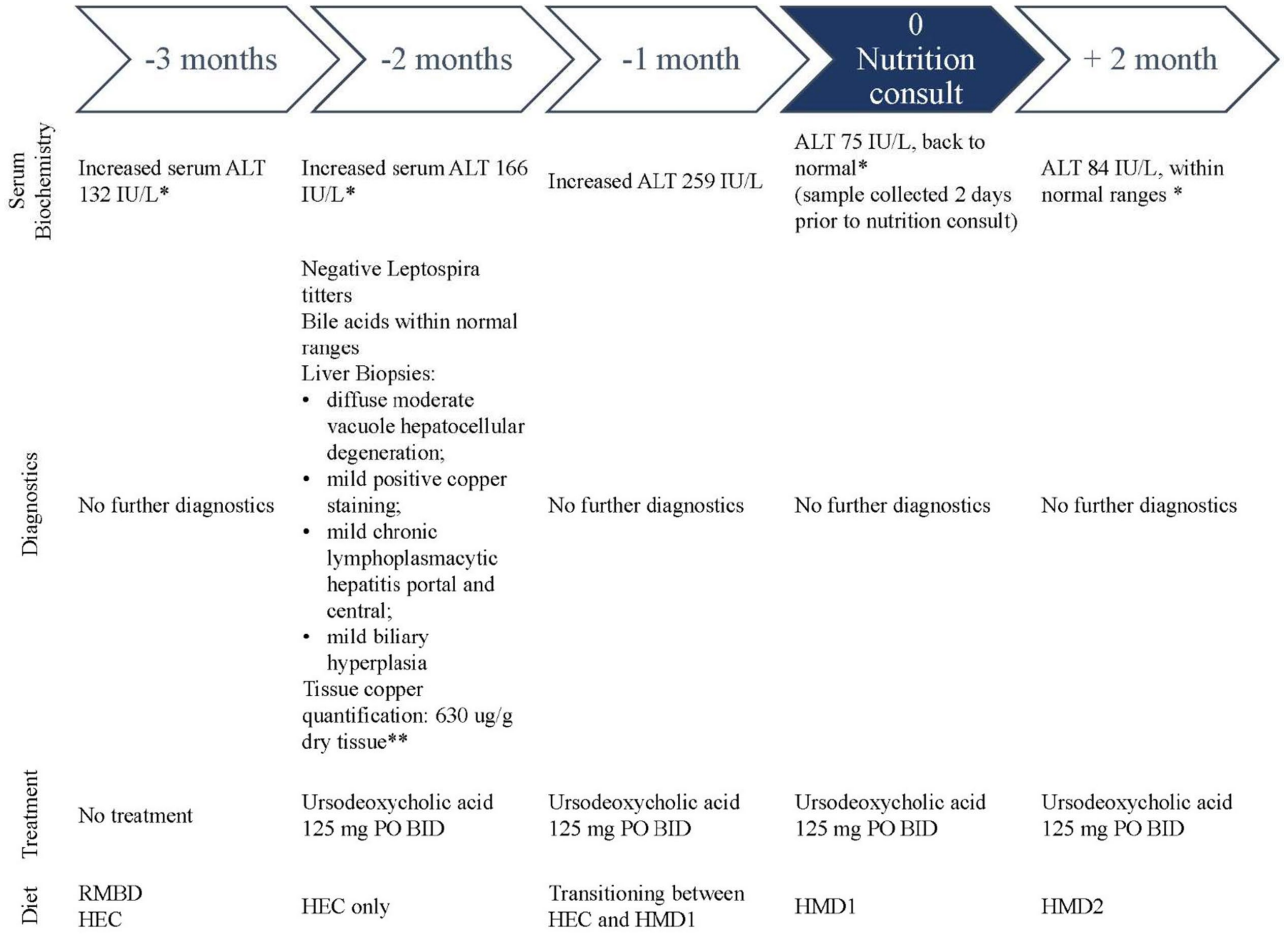
Case history timeline for the nutritional management of a dog with hepatic enzymopathy suspected to be secondary to Cu-associated hepatitis. Each period indicates the biochemical findings, any diagnostics performed, and treatment and diet fed to the patient. The nutrition consult occurred at month 0. Transition between HMD1 and HMD2 occurred over 4 weeks following nutrition consultation ^*^ALT reference range: 18–121 IU/L; ^**^Cu tissue quantification to rule out familial Cu-associated hepatitis: normal <500 μg/g dry tissue (15).

**Figure 2:**
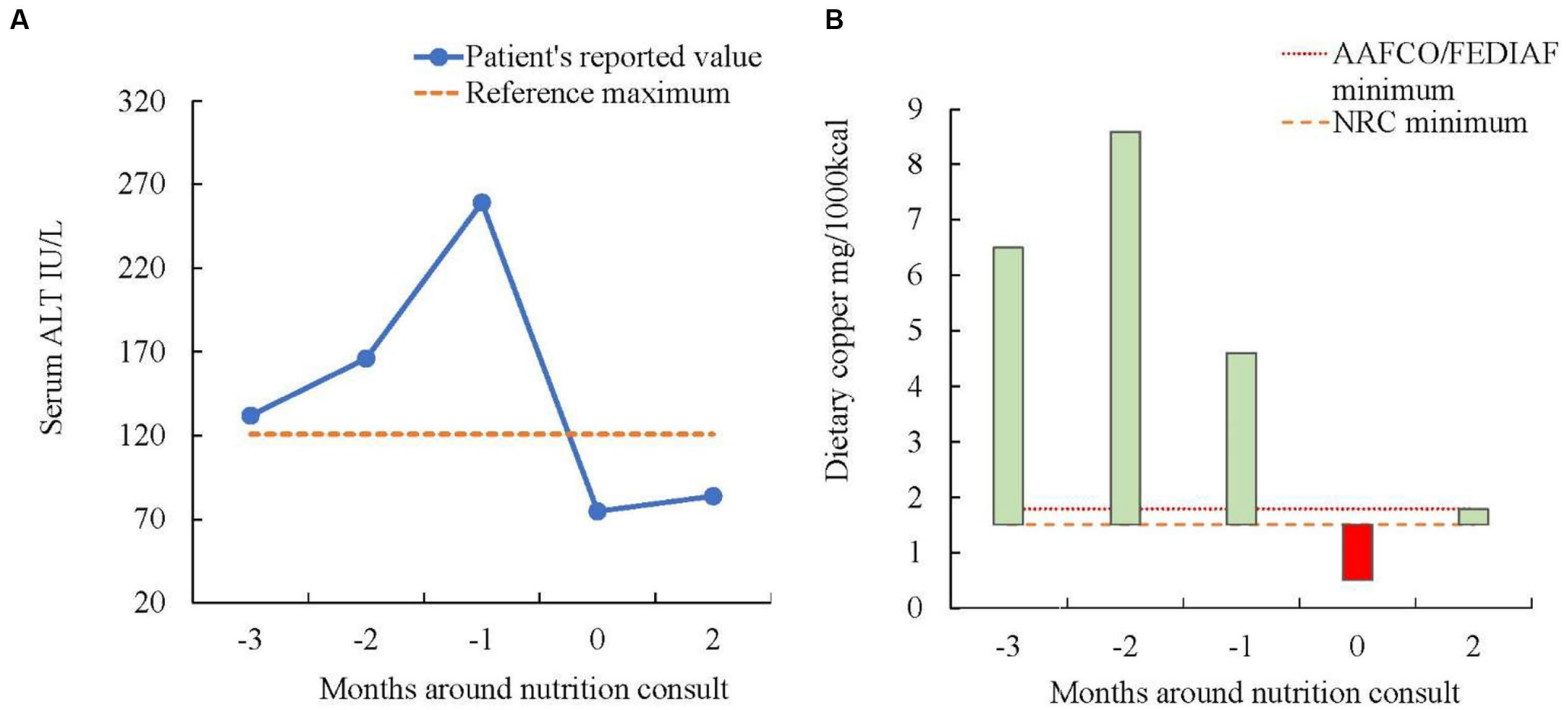
**(A)** Serum ALT concentrations in a 4-year-old, female spayed American Bulldog with hepatic enzymopathy. Nutrition consult occurred at month 0. The patient was eating diets with different copper content. At the time of nutrition consult, the patient was eating an unbalanced diet with copper content below the minimum recommended by NRC, this coincides with the ALT values falling within normal reference ranges. **(B)** This figure exemplifies the dietary Cu content fed to a 4-year-old, female spayed American Bulldog with hepatic enzymopathy compared vs. the minimum requirements by the NRC, AAFCO, and FEDIAF at different time points. The red colored bar indicates when the dietary Cu content was below the minimum recommended by NRC. Nutrition consult occurred at month 0.

A 4-year-old, female spayed American Bulldog presented to the Clinical Nutrition Service at the Ontario Veterinary College’s Health Sciences Center for a homemade diet (HMD) formulation for the nutritional management of hepatic enzymopathy and suspected Cu-associated hepatitis. A case timeline is provided in Figure 1. Medical history obtained from the referring internal medicine specialist revealed a 3-month history of gradual increase in serum ALT activity (Figure 2). On routine bloodwork 3 months before the nutrition consultation, serum ALT activity was found to be increased (132 IU/L; reference range 18–121 IU/L). The following month, a repeated serum biochemistry showed a further increase (166 IU/L). Serum ALT continued to rise over the next month (259 IU/L). No other significant findings or clinical signs were noted during any of these visits. The dog’s owner reported no other ongoing medical concerns for the patient.

To further explore the cause of the increased serum ALT activity, additional diagnostics included: *Leptospira* titers (negative result), bile acids (under normal ranges), and laparoscopic biopsy samples sent for bacterial culture, and Cu staining and quantification. Histopathology of liver samples was consistent with diffuse moderate vacuole hepatocellular degeneration, mild positive Cu staining, mild chronic lymphoplasmacytic hepatitis portal and central, and mild biliary hyperplasia. Cu quantification in liver tissue revealed a slightly higher value (630 μg/g dry tissue) than normal (< 500 μg/g dry tissue) (15). Following biopsy results, the referring internal medicine specialist prescribed ursodeoxycholic acid 125 mg PO BID and temporarily transitioned onto a low-Cu homemade cooked diet (HMD1). The patient was referred to a clinical nutrition consultation for long-term dietary management.

Bloodwork performed 2 days before the nutrition consultation showed serum ALT activity within normal ranges (75 IU/L). The patient ate HMD1 for an 8-week period including a 2-week transition period, 2 weeks as a sole diet, and 4 weeks during the transition onto HMD2. During this time, the patient maintained a stable body weight (BW; 28.2 kg), body condition score (BCS) 5/9 (16), and normal muscle condition score (MCS) (17). A nutritional assessment was performed, and nutrition risk factors were identified using the WSAVA Nutritional Assessment Checklist (18). The risk factors found included multiple pets in the household, previous or ongoing medical conditions, current administration of medications and an unconventional diet. An extended dietary evaluation was pursued using a standard diet history form and a directed inquiry during the consultation.

The patient had been eating a RMBD for more than 1 year before presenting to the internal medicine specialist. Three months before the nutrition consult, at the time of the first blood analyses revealing high serum ALT concentrations, the patient’s diet was mainly RMBD (see text footnote 1) and occasionally HEC (see text footnote 2). When the second blood sample was taken 1 month later, the patient was fed mostly HEC (see text footnote 2); by the third blood sample, 1 month prior to the nutrition consult, the patient was transitioning from HEC (see text footnote 2) to HMD1. The patient’s owner specified that strict sanitary measures were followed for handling and offering RMBD (see text footnote 1) to the patient. After the diagnostics that followed the second blood sample, the patient was recommended a temporary homemade diet (HMD1, Table 1) by the internal medicine specialist while waiting for a nutrition consult. This HMD1 consisted of one full boneless chicken breast (172 g), one cooked steak filet (262 g), and 3/4 cup of potatoes without skin (117 g). Based on this information, the patient’s calorie intake coming from the HMD was estimated to be around 1,074 kcal per day (19). Treats consisted of two commercial pill pockets treats^3^ (23 kcal/treat) and commercial dried fish-based treats^4^ (124 kcal/treat) for a total daily energy intake of around 1,244 kcal.

**Table 1:** Key nutrients of diets fed to a dog with hepatic enzymopathy compared to industry standards.

	Protein (g/1,000 kcal)	Fat (g/1,000 kcal)	Vitamin E (IU/1,000 kcal)	Copper (mg/1,000 kcal)	Zinc (mg/1,000 kcal)
FEDIAF minimum (110 kcal BW^0.75^)	45	13.7	9	1.8 (maximum 2.8 mg/100 gDM)	18
NRC minimum	25	13.8	11.2	1.5	30
AAFCO minimum	45	13.8	12.5	1.8	20
Diet 1: RMBD^a^	122	52	1	4.5	23
Diet 2: HEC^b^	71	71	23.5	8.6	50.3
Diet 3: HMD1	117	45.9	0	0.5	15.9
Diet 4: HMD2	79	45	75	1.8	30.2

^a^Raw meat-based diet.^b^High energy commercial dry food.

The patient’s energy requirements were calculated based on BW, BCS, and MCS using the equations recommended by the 2021 American Animal Hospital Association guidelines (20). The equation 70*BW^0.75^ was used to determine resting energy requirement (RER, 856 kcal/day). Based on an ideal BCS, moderate activity levels, and current energy intake, the initial daily energy requirement (DER) was decided as 1.5 × RER (1,284 kcal).

Using the information provided in the diet history, the Cu levels of the previous diets were assessed and compared to the recommended ranges by NRC, AAFCO, and FEDIAF (1–3). The nutrient analysis was provided by the HEC (see text footnote 2) manufacturer (8.6 mg/1,000 kcal), though the manufacturer of the RMBD (see text footnote 1) failed to provide a nutritional adequacy statement and any nutrient analysis beyond a proximate analysis when this was requested by the Clinical Nutrition Service. Moreover, food samples of this diet were no longer available for nutrient analyses, so the nutrient profile was estimated based on the ingredient list on the product label. Copper level of this diet was estimated using the web-based formulation software BalanceIT® Autobalancer^5^ based on the ingredient list order (Table 1). The Cu content for HMD1 was also estimated using the same software based on the ingredients and amounts provided by the dog’s owner.

According to the medical and diet history, the patient’s problem list consisted of an imbalanced diet and elevated liver enzymes.

### Dietary treatment

Nutritional management of this case was to formulate an HMD as per the owner’s request. The patient’s owner reported having previous experience preparing HMDs for former pets and preferred to feed less processed foods. Following the nutrition consultation, the diet (HMD2) was formulated using the web-based formulation platform, BalanceIT® Autobalancer_,_ which utilizes the USDA database for ingredient nutrient composition. Ingredients were selected in agreement with the owner’s preferences, known palatability for the patient, and consistent availability. Formulation details for HMD2 are available in Supplementary material (Supplementary Table 1 for ingredients and full nutrient profile). Calorie intake considered for the HMD formulation was based on 90% of calculated DER (1,150 kcal), allowing for the remaining 10% (~134 kcal) to be given as treats.

The patient’s owner was instructed to measure the ingredients on a gram scale and was informed that ingredient substitutions will alter the nutrient profile of the diet, therefore, any intended changes would have to be verified and adjusted by the Clinical Nutrition Service. During the consultation and in conversations prior to the formulation, the owner asked if meat ingredients could be given raw. The risks of bacterial contamination were thoroughly explained to the patient’s owner, including the possibility of infection that could lead to further liver damage (21). The dog’s owner was given specific cooking instructions and confirmed that they would follow these instructions.

Given that the patient did not present with any gastrointestinal disease signs or previously reported food intolerance, a standard gradual transition over 7 days was recommended for all the ingredients except for the dietary supplements. A calcium supplement^6^ was recommended to be introduced over the course of 2 weeks, an omega-3 fatty acid source^7^ over 3 weeks, and vitamin/mineral premix^8^ over 4 weeks.

### Monitoring and follow-up

For monitoring, it was suggested to keep a detailed diary to record HMD, treat intake and stool consistency daily, BW and BCS weekly, and inform the Clinical Nutrition Service of any changes. Over the follow-up period, communication with the patient’s owner was limited; however the few times they contacted the Clinical Nutrition Service, they were very positive about how the food transition was going, in terms of palatability, absence of gastrointestinal signs, and recipe compliance. The patient’s BCS and BW remained stable.

Follow-up bloodwork was performed by the family veterinarian 2 months after Clinical Nutrition consultation. All serum biochemistry values were within the normal laboratory range, including serum ALT concentrations (84 IU/L).

## Discussion

This case was referred to the Clinical Nutrition Service with a very comprehensive workup and with absence of clinical signs. Referral to a veterinary nutritionist may be needed for recommendations for Cu-restricted diets given the limited commercial therapeutic diet options with reduced Cu content, which usually involve a moderate protein restriction (12, 22) and was not required for this patient. Furthermore, the patient’s owners had a preference for unconventional diets; therefore, a homemade diet was considered and recommended as an effective alternative. For this patient, hepatic Cu levels fell into the “gray zone” (630 μg/g; 600–1,000 μg/g; rhodamine staining technique) (12), meaning that Cu-associated hepatitis could not be definitively diagnosed. Therefore, the indication of a strict dietary Cu restriction remained uncertain. The commonly used ranges for dietary Cu restriction are based on levels for breed-associated Cu accumulation (23), with dietary intake of Cu below minimum recommendations by AAFCO or NRC (1, 2). The breed of the patient has not been previously reported as predisposed to Cu storage hepatitis (7). There is a main limitation.

Achievement of stable blood liver enzymes within normal ranges was noted in this patient. It is impossible to confirm which factors had a stronger effect on preventing further liver damage, the dietary changes themselves or the combination of dietary management and response to an extended period of ursodeoxycholic acid therapy. Ursodeoxycholic acid administration was consistent after initial prescription by the internal medicine specialist, given its potential properties as choleretic and anti-inflammatory (12). Considering that the Cu content of the previously fed commercial diets was higher than the subsequent HMD, limiting dietary Cu intake was suspected to impact the reduction of serum ALT. The way to confirm these assumptions would be to repeat liver biopsies and Cu quantification, which was not a possibility for a healthy client-owned animal. When the serum ALT activity was reported within normal ranges, the patient was eating HMD1, which was a low copper (0.5 mg/1,000 kcal) yet unbalanced diet, over a period of 8 weeks, including diet transitions. HMD1 presented multiple nutrient deficiencies (Table 1), some of which are considered key nutrients when formulating or choosing diets for liver disease, like Vitamin E and zinc (11). As these hepatoprotective nutrients were limited, there is a strong indication that the reduction of dietary Cu content was the main dietary factor to reduce the hepatic damage. ALT activity is considered the earliest indicator of chronic hepatitis and is also treated as the most important monitoring factor for treatment success (12). The patient of this case report presented improvement in the ALT activity after being fed a low copper diet, and therefore a moderately low copper diet was maintained for over 2 months. This is graphically exemplified in Figure 2, where (a) represents the ALT reported values and (b) the dietary Cu content. To the authors’ knowledge, this is the first time, a case is reported using a low-Cu homemade diet to manage a canine patient in the “gray zone” (12) of hepatic Cu concentration.

Blood liver enzymes remained stable and within normal range after implementation of a nutritionist-designed diet plan. The food recommended and used in this case report, HMD2, was specifically formulated for mild inflammatory hepatobiliary disease. In addition to limited Cu intake, HMD2 also addressed other key nutritional factors considered as per hepatobiliary disease standard of care (11). Although HMD entails high labor intensity for the pet owners, it also provides the nutritionist with a unique opportunity for customization of the nutrient profile.

Protein in the diet should be of good quality and provided in sufficient amounts to support liver regeneration (24). Patient liver function was not compromised, and they did not seem at the risk of developing hepatic encephalopathy or hyperammonaemia (25, 26); therefore, protein levels were not restricted. However, the patient had been fed a high protein diet for a long time so a reduction in protein intake was elected. The target protein levels of HMD2 were between 75 and 80 g/1,000 kcal, in comparison for patients with compromised liver function or hepatic encephalopathy, for whom the target range is between 37 and 50 g/1,000 kcal (11).

As the pathology report identified an inflammation in the evaluated tissue, when formulating HMD2, an enhanced dose of the omega-3 fatty acids eicosapentaenoic acid (EPA) and docosahexaenoic acid (DHA) was included due to their anti-inflammatory properties. The target anti-inflammatory dose was 125 mg/kgBW^0.75^ (~1,500 mg/day) of EPA and DHA per day (27–29). The introduction of the concentrated supplement (see text footnote 7) was recommended to start after all other ingredients of the diet were already known to be well tolerated.

Excessive Cu intake was avoided to prevent further accumulation in the liver tissue, which could lead to increased oxidative damage. From the biopsy results and Cu quantification, genetic Cu storage disease was ruled out (15), suggesting that the mild Cu accumulation seen was secondary to hepatobiliary inflammation. Therefore, a strict Cu-restricted diet was not required and only a mild restriction of Cu was considered for the formulation of diet HMD2. A strict Cu-restricted diet is recommended, as for confirmed genetic Cu storage disease, Cu levels should be under 1.25 mg/1,000 kcal (11, 23). The target values for HMD2 of this mineral were just above the low end recommended by AAFCO (1.83 mg/1,000 kcal) (2) and the minimum required by the NRC (1.5 mg/1,000 kcal) (1). In comparison, one study found that the median concentration in commercial dog maintenance foods was 4.4 mg/1,000 kcal (22, 30), with only the liver-targeted therapeutic diets going below 2.1 mg/1,000 kcal (11, 23). The possibility of adjusting the Cu levels of the diet further by changing to a Cu-restricted vitamin/mineral supplement was discussed with the patient’s owner and left open, pending bloodwork results. In this case, Cu levels of the diets varied between 8.6 and 0.5 mg/kcal. The estimated Cu content of the RMBD (see text footnote 1) was 4.5 mg/kcal, which is close to the average values for commercial diets previously reported in the literature (22, 30). However, given that the ingredient list for the RMBD (see text footnote 1) references organ meats like liver and kidney meat, the Cu content could be even higher than estimated, as they are considered ingredients with a high concentration of Cu (31).

Alternatives or additional recommendations to dietary Cu restriction would have been dietary zinc supplementation (5–10 mg/kg BW or > 50 mg/1,000 kcal) (7, 11). Although it has been reported that supplementation of zinc gluconate did not produce any difference for Cu-associated hepatitis in Labradors (9). The prescription of *D*-Penicillamine has also been reported as an option to reduce Cu availability (7, 11). In this case, the dog owner preferred to try a low Cu diet first before the addition of extra medications. Furthermore, the efficacy of this medication without a Cu-restricted diet may be reduced (12).

Vitamin E, as an antioxidant, could potentially help with some forms of acute and chronic liver injuries by reducing the oxidative damage caused by free radicals and reactive oxygen (11). Vitamin E content of HMD2 was formulated at 75 IU/1,000 kcal, greatly exceeding minimum requirements by AAFCO (12.5 IU/1,000 kcal) (2, 3) or NRC (8.9 IU/1,000 kcal) (1). Vitamin E recommended contents for hepatobiliary disease are above 100 IU/1,000 kcal (11). Further supplementation of vitamin E was left as an open option if the initial recommendations failed in the goal of maintaining serum liver enzymes within normal ranges.

The patient was eating four different diets, all of which presented different nutrient profiles and contrasting levels of Cu. Blood ALT values seemed to increase when dietary Cu was higher and returned to normal ranges when the Cu was close or under the minimum requirement established by the NRC (1). This patient was presented to the OVC Clinical Nutrition service for a formulation of an HMD due to difficulty in finding a diet with low Cu levels without restriction of protein content. Furthermore, it is challenging for pet owners and veterinarians to find commercial diets moderate in Cu content, as most greatly exceed the minimum requirements (22, 30). Even gastrointestinal therapeutic diets are not moderate in Cu content, despite gastrointestinal tract health and Cu excretion being so closely related (5, 32). To the author’s knowledge, there are no reports of recommended Cu levels for secondary Cu accumulation in the liver. Thus, further studies in which a reduction of Cu intake is the only nutrient intervention are warranted to determine standard ranges for dog maintenance diets.

## Conclusion

This case report exemplifies the need for establishing different grades of dietary Cu restriction when mild or subclinical liver disease is present and in the absence of genetic predisposition. Biochemical analysis of liver enzymes was used for monitoring hepatocellular integrity. The elevated ALT values were the trigger for further diagnostics and therapeutic interventions. It also highlights the utility of an HMD and demonstrates the benefits of an individualized approach in the absence of a commercial diet alternative for patients that may benefit from Cu restriction and may not need the remaining limitations of commercial therapeutic diets targeted to liver disease. It is important to mention that this is only one case subject and further study of similar cases is warranted to confirm any clinical inferences.

Further research is warranted to determine a safe upper limit of Cu to be included in the nutritional guidelines for commercially available foods, especially with the rise of pet foods that use organ meat as the main ingredients.

## Data availability statement

The original contributions presented in the study are included in the article/Supplementary material, further inquiries can be directed to the corresponding author.

## Ethics statement

Ethical review and approval were not required for the animal study because this was a retrospective case report. Written informed consent was obtained from the owners for the participation of their animals in this study.

## Author contributions

FP was the clinician in charge of management of case. CG was the supervising faculty during management of case. FP, CG, ÉR, and AV participated in collection of data, writing and editing manuscript, and review of final submission. All authors contributed to the article and approved the submitted version.

## Footnotes

^1^Beef meal – K9 Kitchen, Brantford, Ontario, Canada.

^2^Inukshuk 32/32—Inukshuk Professional Dog Food, Fredericton, New Brunswick, Canada.

^3^Greenies pill pockets peanut butter flavor capsule. Mars Canada, Bolton, Ontario, Canada.

^4^Icelandic+ Herring Whole Fish Dog Treats, Icelandic PLUS, EHF, Smáratorg, Kópavogur, Iceland.

^5^BalanceIT® Autobalancer software. DVM Consulting, Inc., Woodland, CA, United States.

^6^Tums Ultra Strength 1000. GlaxoSmithKline, Mississauga, Ontario, Canada.

^7^Nordic Naturals Omega 3 pet liquid. Nordic Naturals Mig., Watsonville, California, United States.

^8^Balance IT canine vitamin, mineral and amino acid powder. DVM Consulting, Inc., Woodland, California, United States. Patents US 8,968,806, US 10,194,685 & CA 2,606,109.
