# Supplementary material for: Nutritional management of a dog with hepatic enzymopathy suspected to be secondary to copper-associated hepatitis: a case report
Source: Front Vet Sci. 2023 Dec 11;10:1215447. doi: 10.3389/fvets.2023.1215447 (PMC10749294; doi:10.3389/fvets.2023.1215447)
Supplement: Supplementary file 1 [file Table_1.DOCX]

Supplementary Material

**Nutritional management of a dog with hepatic enzymopathy suspected to be secondary to copper-associated hepatitis: A case report**

**FM Poblanno-Silva^1^, CE Grant^1^, EM Ribeiro^1^, A Verbrugghe^1^***

^1^ Department of Clinical Studies, Ontario Veterinary College, University of Guelph, Ontario, Canada.

*** Correspondence:** [averbrug@uoguelph.ca](mailto:averbrug@uoguelph.ca)

**Supplementary Table 1:** Ingredient list and nutrient profile of a nutritionist-formulated homemade diet (HMD2) for a dog with hepatic enzymopathy suspected to be secondary to copper-associated hepatitis compared to AAFCO minimum requirements for adult dog maintenance.

| **Ingredient** | | **Amount** | | |
| --- | --- | --- | --- | --- |
| Chicken breast, skinless, cooked without oil | | 300 g | | |
| Egg cooked hard-boiled | | 100 g (~ 2 large eggs) | | |
| White rice, long grain | | 150 g | | |
| White potato, baked with skin | | 100 g | | |
| Canned pumpkin | | 212 g | | |
| Canola oil | | ½ tsp | | |
| Tums™ Ultra Strength 1000^e^ | | 2 tablets | | |
| Nordic Naturals™ Omega 3 pet liquid^f^ | | 6 ml | | |
| BalanceIT® canine vitamin, mineral and amino acid powder supplement^g^ | | 10 g | | |
| **Nutrient** | **Units** | **Adult Minimum** | **HMD1** | **HMD2** |
| Crude protein | g/1000kcal | 45 | 117 | 79.1 |
| Arginine | g/1000kcal | 1.28 | 7 | 5 |
| Histidine | g/1000kcal | 0.48 | 3.8 | 2.2 |
| Isoleucine | g/1000kcal | 0.95 | 5.6 | 3.6 |
| Leucine | g/1000kcal | 1.7 | 9 | 6.1 |
| Lysine | g/1000kcal | 1.58 | 9.8 | 6.3 |
| Methionine | g/1000kcal | 0.83 | 3.09 | 2 |
| Methionine-cystine | g/1000kcal | 1.63 | 4.4 | 3 |
| Phenylalanine | g/1000kcal | 1.13 | 4.6 | 3.3 |
| Phenylalanine-tyrosine | g/1000kcal | 1.85 | 8.5 | 6.1 |
| Threonine | g/1000kcal | 1.2 | 5 | 3.3 |
| Tryptophan | g/1000kcal | 0.4 | 1.3 | 0.8 |
| Valine | g/1000kcal | 1.23 | 5.7 | 3.9 |
| Crude Fat | g/1000kcal | 13.8 | 45.9 | 45.5 |
| Linoleic Acid | g/1000kcal | 2.8 | 1.85 | 6.2 |
| alpha-Linoleic Acid | g/1000kcal | ND | ND | ND |
| Eicosapentanoic + Docosahexaenoic Acid | g/1000kcal | ND | 0.048 | 1.3 |
| **Minerals** |  |  |  |  |
| Calcium | g/1000kcal | 1.25 | 0.047 | 2.15 |
| Phosphorus | g/1000kcal | 1 | 1.03 | 1.64 |
| Ca:P ratio |  | 1:1 | 1:0.05 | 1.3:1 |
| Potassium | g/1000kcal | 1.5 | 1.59 | 3.60 |
| Sodium | g/1000kcal | 0.2 | 0.29 | 0.41 |
| Chloride | g/1000kcal | 0.3 | 0.39 | 0.69 |
| Magnesium | g/1000kcal | 0.15 | 0.12 | 0.23 |
| Iron | mg/1000kcal | 10 | 10.12 | 22.40 |
| Copper | mg/1000kcal | 1.83 | 0.55 | 1.88 |
| Manganese | mg/1000kcal | 1.25 | 0.22 | 2.10 |
| Zinc | mg/1000kcal | 20 | 15.90 | 30.20 |
| Iodine | mg/1000kcal | 0.25 | 0.00 | 0.35 |
| Selenium | mg/1000kcal | 0.08 | 0.12 | 0.10 |
| **Vitamins and Others** |  |  |  |  |
| Vitamin A | IU/1000kcal | 1250 | 32 | 6263.3 |
| Vitamin D | IU/1000kcal | 125 | 21 | 161 |
| Vitamin E | IU/1000kcal | 12.5 | 2.12 | 75 |
| Thiamine | mg/1000kcal | 0.56 | 0.56 | 0.95 |
| Riboflavin | mg/1000kcal | 1.3 | 0.64 | 2.1 |
| Pantothenic Acid | mg/1000kcal | 3 | 3 | 7.7 |
| Niacin | mg/1000kcal | 3.4 | 34 | 24.63 |
| Pyridoxine | mg/1000kcal | 0.38 | 2.1 | 2.1 |
| Folic Acid | mg/1000kcal | 0.054 | 0.038 | 0.277 |
| Vitamin B12 | mg/1000kcal | 0.007 | 0.009 | 0.007 |
| Choline | mg/1000kcal | 340 | 102 | 596 |
