# Supplementary material for: Nutritional management of a dog with hepatic enzymopathy suspected to be secondary to copper-associated hepatitis: a case report
Source: Front Vet Sci. 2023 Dec 11;10:1215447. doi: 10.3389/fvets.2023.1215447 (PMC10749294; doi:10.3389/fvets.2023.1215447)
Supplement: Supplementary file 2 [file Data_Sheet_1.PDF]

CARE
case report guidelines

CARE Checklist of information to include when writing a case report

(cc) BY-NC-ND

Topic	Item	Checklist item description	Reported on Line
**Title**	**1**	The diagnosis or intervention of primary focus followed by the words "case report"	1-2
**Key Words**	**2**	2 to 5 key words that identify diagnoses or interventions in this case report, including "case report"	6-7
**Abstract (no references)**	**3a**	Introduction: What is unique about this case and what does it add to the scientific literature?	15-18
	**3b**	Main symptoms and/or important clinical findings	11-17
	**3c**	The main diagnoses, therapeutic interventions, and outcomes	22-30
	**3d**	Conclusion—What is the main "take-away" lesson(s) from this case?	30-34
**Introduction**	**4**	One or two paragraphs summarizing why this case is unique (**may include references**)	36-64
**Patient Information**	**5a**	De-identified patient specific information.	66
	**5b**	Primary concerns and symptoms of the patient.	68-75
	**5c**	Medical, family, and psycho-social history including relevant genetic information	76-82
	**5d**	Relevant past interventions with outcomes	82-85
**Clinical Findings**	**6**	Describe significant physical examination (PE) and important clinical findings.	86-94
**Timeline**	**7**	Historical and current information from this episode of care organized as a timeline	69 - Figure 1
**Diagnostic Assessment**	**8a**	Diagnostic testing (such as PE, laboratory testing, imaging, surveys).	71-82
	**8b**	Diagnostic challenges (such as access to testing, financial, or cultural)	95-106
	**8c**	Diagnosis (including other diagnoses considered)	78-123
	**8d**	Prognosis (such as staging in oncology) where applicable	n/a
**Therapeutic Intervention**	**9a**	Types of therapeutic intervention (such as pharmacologic, surgical, preventive, self-care)	125
	**9b**	Administration of therapeutic intervention (such as dosage, strength, duration)	table 1
	**9c**	Changes in therapeutic intervention (with rationale)	125-145
**Follow-up and Outcomes**	**10a**	Clinician and patient-assessed outcomes (if available)	147-141
	**10b**	Important follow-up diagnostic and other test results	153-155
	**10c**	Intervention adherence and tolerability (How was this assessed?)	149-152
	**10d**	Adverse and unanticipated events	n/a
**Discussion**	**11a**	A scientific discussion of the strengths AND limitations associated with this case report	157-165
	**11b**	Discussion of the relevant medical literature **with references**.	199-216
	**11c**	The scientific rationale for any conclusions (including assessment of possible causes)	231-239
	**11d**	The primary "take-away" lessons of this case report (without references) in a one paragraph conclusion	240-242
**Patient Perspective**	**12**	The patient should share their perspective in one to two paragraphs on the treatment(s) they received	n/a
**Informed Consent**	**13**	Did the patient give informed consent? Please provide if requested	**Yes** ☐ x **No** ☐
